# Risk factors for thyroid nodules in a health examination population: a cross-sectional study and development of a simplified predictive model

**DOI:** 10.3389/fendo.2025.1738544

**Published:** 2026-01-15

**Authors:** Hangtian Yu, Jingle Cao, Jing Han, Yang Li, Wenyu Li, Zihan Li, Jinjia Zhang, YaLi Zhang

**Affiliations:** General Medical Department, The Second Hospital of Hebei Medical University, Shijiazhuang, Hebei, China

**Keywords:** body mass index, health examination, metabolic indicators, risk factors, thyroid nodules

## Abstract

**Background:**

Thyroid nodules (TNs) are common in adults and have been linked to various metabolic and demographic factors. This study aims to explore the associations between metabolic indicators and TNs in a Chinese health examination population, and to develop a simplified predictive model based on independent risk factors.

**Methods:**

We conducted a cross-sectional analysis of 23,305 adults (12,977 men, 10,328 women; aged 18–90 years) who underwent health examinations at the Second Hospital of Hebei Medical University between January 2021 and December 2022. Exclusion criteria included prior thyroid surgery, endocrine or systemic disorders, pregnancy, and incomplete data. Demographic, lifestyle, and biochemical parameters were collected. Group differences were assessed using chi-square tests for categorical variables and t-tests or Mann-Whitney U tests for continuous variables. Univariate and multivariate logistic regression analyses were performed to identify independent risk factors, with model performance evaluated by the area under the receiver operating characteristic curve (AUC).

**Results:**

The overall prevalence of TNs was 64.7% (n=15,085). The prevalence increased from 38.8% in those aged 30 years or younger to 87.8% in those older than 70 years (P for trend <0.01), and was higher in women (70.8%) compared to men (59.9%) (χ²=509.8, P<0.01). In multivariate analysis, older age (OR = 1.06 per year, 95% CI: 1.06–1.06, P<0.01), female sex (OR = 2.12, 95% CI: 1.93–2.32, P<0.01), and higher body mass index (OR = 1.04 per unit, 95% CI: 1.03–1.05, P<0.01) were identified as independent risk factors. The three-variable model yielded an AUC of 0.706.

**Conclusions:**

Thyroid nodules are highly prevalent in this health examination population. Age, female sex, and higher body mass index are independent risk factors. Other metabolic disturbances were more common in individuals with TNs, but they were not independent predictors. A simplified model based on age, sex, and body mass index may help identify high-risk individuals in large-scale screenings.

## 1 Introduction

Thyroid nodules (TNs) are among the most common endocrine disorders. With the widespread use of high-resolution ultrasonography, their detection rate has risen sharply, making them one of the most frequently identified findings during routine health examinations (1). Recent studies estimate that TNs affect more than half of the adult population, although the majority of thyroid nodules are benign, a subset carries a risk of malignancy (2–4). Therefore, early identification of high-risk individuals is crucial for effective prevention and intervention.

In recent years, increasing attention has been directed toward the role of metabolic syndrome and lifestyle factors in the development of TNs. Metabolic syndrome is a pathological condition characterized by central obesity, insulin resistance, hypertension, and dyslipidemia, and is closely associated with both endocrine and cardiovascular disorders. Several studies have reported associations between metabolic syndrome components, including obesity, dyslipidemia, hyperglycemia, and hyperuricemia, are associated with an increased risk of TNs (5–9). Lifestyle behaviors such as smoking and alcohol consumption have also been linked to TNs (10). However, findings across different populations and regions remain inconsistent, and the specific contributions of individual factors are still not fully understood. Moreover, many existing studies are limited by small sample sizes, insufficient evaluation of metabolic variables, and inadequate control of potential confounders.

To address these gaps, we conducted a large-scale cross-sectional study based on 23,305 adults undergoing physical examinations in northern China. We aimed to: (1) assess the prevalence of TNs in a large adult population undergoing routine health screening; (2) explore their associations with a wide spectrum of metabolic factors using rigorous statistical methods; and (3) construct a supplementary logistic regression-based prediction model as a secondary exploratory analysis. Our findings are intended to enrich the epidemiological understanding of TNs and support refined screening strategies in general health management.

## 2 Methods

### 2.1 Study population

This cross-sectional study included adults aged 18–90 years who underwent routine health examinations at the Health Management Center of the Second Hospital of Hebei Medical University between January 2021 and December 2022. A total of 68,834 records were initially reviewed. After excluding individuals with missing key data, extreme outliers, a history of thyroid surgery, known thyroid disease, systemic or endocrine disorders, or pregnancy, 23,305 participants were included in the final analysis. All data were anonymized before analysis. The study adhered to the principles of the Declaration of Helsinki and was approved by the institutional ethics committee.

### 2.2 Data collection

All participants completed standardized questionnaires and underwent anthropometric measurements and laboratory tests. Height and weight were measured with participants wearing light clothing and no shoes. Body mass index (BMI) was calculated as weight in kilograms divided by the square of height in meters. Blood pressure (BP) was measured from the right brachial artery after at least 5 minutes of rest using an electronic sphygmomanometer.

After an overnight fast of 8 to 10 hours, venous blood samples were collected in the morning. Samples were centrifuged within 1 hour and analyzed within 1 to 3 hours in the hospital’s central clinical laboratory following standardized procedures. Fasting blood glucose (FBG) was determined using the glucose oxidase-peroxidase method. Serum lipid profiles, including triglycerides (TG), total cholesterol (TC), low-density lipoprotein cholesterol (LDL-C), and high-density lipoprotein cholesterol (HDL-C), were measured using enzymatic colorimetric assays. Serum uric acid (UA) was assessed by the uricase-peroxidase method, serum creatinine (SCR) was measured using the sarcosine oxidase method, and homocysteine (HCY) levels were quantified via an enzyme cycling assay.

Thyroid ultrasonography was performed by experienced sonographers using standardized protocols. Participants were examined in the supine position with the neck extended. The thyroid gland was scanned in both transverse and longitudinal planes to assess its size, echotexture, and the presence of any nodules. Thyroid nodules were identified as discrete lesions within the thyroid parenchyma that were distinguishable from the surrounding tissue.

### 2.3 Definition of variables

BMI was categorized based on Chinese adult criteria: underweight (<18.5 kg/m²), normal (18.5–23.9 kg/m²), overweight (24–27.9 kg/m²), and obese (≥28 kg/m²) (11). BP was defined as hypotension (<90/60 mmHg), normal (90–139/60–89 mmHg), or hypertension (≥140/90 mmHg) (12). Abnormal metabolic indicators were defined as follows: FBG ≥6.1 mmol/L, HCY >15 µmol/L, SCR above sex-specific reference ranges (≥133 µmol/L for males and ≥115 µmol/L for females), UA ≥420 µmol/L for males or ≥360 µmol/L for females, TC ≥5.2 mmol/L, TG ≥1.7 mmol/L, HDL-C <1.0 mmol/L for males or <1.3 mmol/L for females, and LDL-C ≥3.4 mmol/L (13–18). Smoking and alcohol consumption were self-reported and categorized as “yes” if participants reported either occasional or regular use; otherwise, they were classified as “no.” TNs status were determined based on ultrasound results. Participants with unilateral or bilateral TNs were assigned to the TN group, while those without nodules were assigned to the Non-TN group.

### 2.4 Statistical analysis

First, the demographic characteristics of the total cohort and sex-specific subgroups were summarized. Continuous variables (e.g., age, BMI, blood pressure) with normal distributions were expressed as mean ± standard deviation (SD) and compared using independent t-tests. When the assumption of variance homogeneity was met, comparisons between males and females were performed using Student’s t-test; otherwise, Welch’s t-test was applied. For variables that did not meet normality assumptions, the Wilcoxon rank-sum test was used. Categorical variables (e.g., smoking/drinking status, TNs classification) were presented as frequencies (percentages) and compared using Pearson’s chi-square (χ²) test (19).

Second, TN prevalence was analyzed across six age strata (≤30, >30–40, >40–50, >50–60, >60–70, >70 years), stratified by sex. TN prevalence rates were calculated for each subgroup, and intergroup comparisons were performed using χ² tests. In addition, Cochran–Armitage trend tests were conducted to assess age-related trends in TN prevalence, accounting for the ordinal nature of age strata (20).

Third, clinical and metabolic characteristics were compared between the TN and Non-TN groups, both in the overall population and stratified by sex. Comparisons were based on predefined categorical thresholds for each variable (e.g., BMI category, BP status, glucose, and lipid levels) using chi-square tests.

Fourth, logistic regression analyses were conducted to identify risk factors associated with TNs. Univariate logistic regression was performed to evaluate the crude association between each variable and TNs presence, generating odds ratios (OR) and 95% confidence intervals (CI). Variables included in the multivariable logistic regression model were selected based on a combination of statistical significance in univariate analyses and a priori clinical relevance, rather than relying solely on data-driven selection, as commonly adopted in observational studies (9, 21).

Finally, we developed a clinical prediction model incorporating candidate variables selected a priori based on clinical relevance and prior literature, and evaluated their independent associations using multivariable logistic regression. The dataset was then randomly divided into a training set (70%) for model development and a validation set (30%) for performance evaluation. Multivariable logistic regression was performed in the training set to construct the prediction model, and ORs with 95% CIs were calculated for each predictor. Predicted probabilities of the outcome were generated for all participants. For the prediction model, model performance was evaluated from both discrimination and calibration perspectives. Model discrimination was evaluated using the area under the receiver operating characteristic curve (AUC-ROC), and calibration was assessed by Hosmer–Lemeshow goodness-of-fit test and calibration curves to examine the agreement between predicted and observed probabilities (22, 23).

## 3 Results

### 3.1 Baseline characteristics of the study population by sex

A total of 23,305 adults (12,977 men and 10,328 women) were included. The mean age was 51.36 ± 13.29 years, significantly higher in men (52 ± 14 years) than women (50 ± 13 years; t = 12.13, P < 0.01). Mean BMI was 25.16 ± 3.42 kg/m², higher in men (26.2 ± 3.2) than women (23.9 ± 3.3; t = 52.64, P < 0.01). SBP and DBP were also higher in men (134 ± 17/83 ± 11 mmHg) than in women (125 ± 19/75 ± 11 mmHg; both P < 0.01). Smoking (27% vs. 0.03%) and alcohol use (52% vs. 0.44%) were far more prevalent in men than women (both χ² > 3,000, P < 0.01) (refer **Table 1**).

**Table 1:** Demographic and clinical characteristics of participants stratified by sex.

Characteristics	Total (n = 23,305)	Male (n = 12,977)	Female (n = 10,328)	t/χ² -value	P- value
Age(years)	51.36 ± 13.29	52 ± 14	50 ± 13	t=12.13	<0.01
BMI(kg/m^2^)	25.16 ± 3.42	26.2 ± 3.2	23.9 ± 3.3	t=52.64	<0.01
SBP(mmHg)	129.93 ± 18.46	134 ± 17	125 ± 19	t=34.84	<0.01
DBP(mmHg)	79.06 ± 11.61	83 ± 11	75 ± 11	t=54.79	<0.01
Smoking (%)					
No	19,787(84.90)	9,462 (73)	10,325 (99.97)	χ²=7,389.02	<0.01
Yes	3,518 (15.10)	3,515 (27)	3 (0.03)		
Alcohol use (%)					
No	16,520(70.89)	6,237 (48)	10,283 (99.56)	χ²=3,282.88	<0.01
Yes	6,785 (29.11)	6,740 (52)	45 (0.44)		
TN (%)					
Negative	8,220(35.27)	5,203 (40)	3,017 (29)	χ²=7,211.45	<0.01
Positive - Unilateral	6,882(29.53)	3,781 (29)	3,101 (30)		
Positive - Bilateral	8,203(35.20)	3,993 (31)	4,210 (41)		

Data are presented as mean ± SD for normally distributed continuous variables and as number (percentage) for categorical variables. Group comparisons were performed using independent t-tests for continuous variables and Pearson’s chi-square (χ²) tests for categorical variables.

### 3.2 Age- and sex-specific prevalence of thyroid nodules

The prevalence of TNs in the study population increased progressively with age. Overall, 64.73% of participants (15,085 of 23,305) had TNs. Among those aged ≤30 years, TN prevalence was 38.83%, rising to 45.72% in the 31–40 age group, 59.84% in the 41–50 age group, 71.50% in the 51–60 age group, 80.90% in the 61–70 age group, and reaching 87.79% in participants over 70 years (χ² for trend = 2,071.27, P < 0.01). Cochran–Armitage trend tests confirmed a significant increasing trend in TN prevalence with age in the overall population (Z = 45.11, P < 0.001), as well as separately in women (Z = 27.90, P < 0.001) and men (Z = 37.60, P < 0.001).

Women consistently exhibited higher TN prevalence than men across all age groups. In the youngest group (≤30 years), 46.04% of women had TNs compared with 36.57% of men. This sex difference remained evident in all older age groups, with the prevalence reaching 91.02% in women and 85.86% in men over 70 years. Overall, 70.79% of women had TNs compared with 59.91% of men, a statistically significant difference (χ² = 297.82, P < 0.01). Detailed age- and sex-specific prevalence is presented in **Table 2**.

**Table 2:** Age- and sex-specific prevalence of thyroid nodules.

Age	Total		Male		Female		Male vs female	
	Number	TN prevalence (%)	Number	TN prevalence (%)	Number	TN prevalence (%)	χ²-value	P- value
≤30	582	226(38.83)	443	162(36.57)	139	64(46.04)	3.61	0.57
>30~≤40	5184	2,370(45.72)	2511	943(37.55)	2,673	1,427(53.39)	130.12	<0.01
>40~≤50	6235	3,731(59.84)	3210	1,639(51.06)	3,025	2,092(69.16)	211.50	<0.01
>50~≤60	5729	4,096(71.5)	3330	2,214(66.49)	2,399	1,882(78.45)	97.34	<0.01
>60~≤70	3372	2,782(80.9)	2104	1,632(77.57)	1,268	1,096(86.44)	39.70	<0.01
>70	2203	1,734(87.79)	1379	1,184(85.86)	824	750(91.02)	12.34	<0.01
Total	23305	15,085(64.73)	12,977	7,774(59.91)	10,328	7,311(70.79)	297.82	<0.01
χ² -value		2,071.27		147.30		817.85		
P- value		<0.01		<0.01		<0.01		
Trend test	Z = 45.11, P < 0.001		Z = 37.60, P < 0.001		Z = 27.90, P < 0.001			

TNs prevalence is expressed as a number and percentage within each age and sex subgroup. Between-group comparisons were assessed using Pearson’s chi-square test. A trend test for age-related changes in TN prevalence was performed using the Cochran-Armitage trend test.

### 3.3 Clinical and metabolic characteristics by thyroid nodule status

When participants were divided by TN status, several clinical and metabolic differences were observed. In the TN group, the prevalence of overweight/obesity was higher than in the Non-TN group (χ² = 8.48, P = 0.04), as were rates of hypertension (χ² = 208.55, P < 0.01) and abnormal FBG (χ² = 164.02, P < 0.01).

Biochemical abnormalities were also more frequent in the TN group. Elevated UA and SCR were significantly more common among TN-positive participants (all P < 0.01), and in women, elevated TG levels were additionally associated with TN presence (P < 0.01).

Sex-specific analyses highlighted lifestyle differences. Among men, smoking and alcohol use were significantly associated with TN occurrence (χ² = 26.12 and 18.25, respectively; both P < 0.01). In contrast, these associations could not be reliably assessed in women due to the extremely low prevalence of smoking and alcohol use. Detailed comparisons of clinical and metabolic characteristics between TN and Non-TN groups are presented in **Table 3**.

**Table 3:** Clinical and metabolic profiles of participants with and without thyroid nodules.

Variate	Total (n=23,305)				Male (n=12,977)				Female (n=10,328)			
	TN	Non-TN	χ²-value	P- value	TN	Non-TN	χ²-value	P- value	TN	Non-TN	χ²-value	P- value
Smoking			2.54	0.11			26.12	<0.01			–	–
Yes	2,235	1,283			2,233	1,282			2	1		
No	12,850	6,937			5,541	3,921			7,309	3,016		
Alcohol use			179.8	<0.01			18.25	<0.01			–	–
Yes	3,947	2,838			3,918	2,822			29	16		
No	11,138	5,382			3,856	2,381			7,282	3,001		
BMI			8.48	0.04			16.07	<0.01			91.63	<0.01
Underweight	205	133			35	37			170	96		
Normal	5,399	3,048			1,753	1,263			3,646	1,785		
Overweight	6,646	3,582			3,989	2,698			2,657	884		
Obese	2,835	1,457			1,997	1,205			838	252		
BP			208.55	<0.01			168.85	<0.01			190.2	<0.01
Hypotension	520	276			98	60			422	216		
Normal	3,715	2,549			1,305	1,238			2,410	1,311		
Hypertension	5,096	2,064			3,337	1,665			1,759	399		
FBG			164.02	<0.01			136.87	<0.01			94.07	<0.01
Normal	12,405	7,283			5,987	4,441			6,418	2,842		
Abnormal	2,680	937			1,787	762			893	175		
HCY			3.19	0.07			36.38	<0.01			31.97	<0.01
Normal	11,110	6,143			4,575	3,337			6,535	2,806		
Abnormal	3,975	2,077			3,199	1,866			776	211		
SCR			31.7	<0.01			39.57	<0.01			16.27	<0.01
Normal	14,695	8,101			7,437	5,086			7,258	3,015		
Abnormal	390	119			337	117			53	2		
UA			31.22	<0.01			46.74	<0.01			21.9	<0.01
Normal	12,300	6,452			6,029	3,760			6,271	2,692		
Abnormal	2,785	1,768			1,745	1,443			1,040	325		
TC												
Normal	11,931	6,584	3.23	0.07	6,392	4,144	13.38	<0.01	5,539	2,440	31.48	<0.01
Abnormal	3,154	1,636			1,382	1,059			1,772	577		
TG			1.42	0.23			4.75	0.03			43.83	<0.01
Normal	10,770	5,807			5,021	3,262			5,749	2,545		
Abnormal	4,315	2,413			2,753	1,941			1562	472		
HDL-C			1.67	0.2			1.26	0.26	,		3.4	0.06
Normal	13,648	7,393			6,624	4,471			7,024	2,922		
Abnormal	1,437	827			1,150	732			287	95		
LDL-C			1.85	0.17			12.72	<0.01			30.96	<0.01
Normal	11,312	6,231			6,020	3,887			5,292	2,344		
Abnormal	3,773	1,989			1,754	1,316			2,019	673		

Categorical variables were compared between groups using Pearson’s chi-square test. The TN group includes participants diagnosed with unilateral or bilateral thyroid nodules. Metabolic indicators were categorized based on standard clinical thresholds (see Methods). Smoking and alcohol use were not analyzed in females due to their extremely low prevalence in this subgroup, which precludes reliable evaluation of associations.

### 3.4 Independent risk factors identified by logistic regression

**Figure 1:**
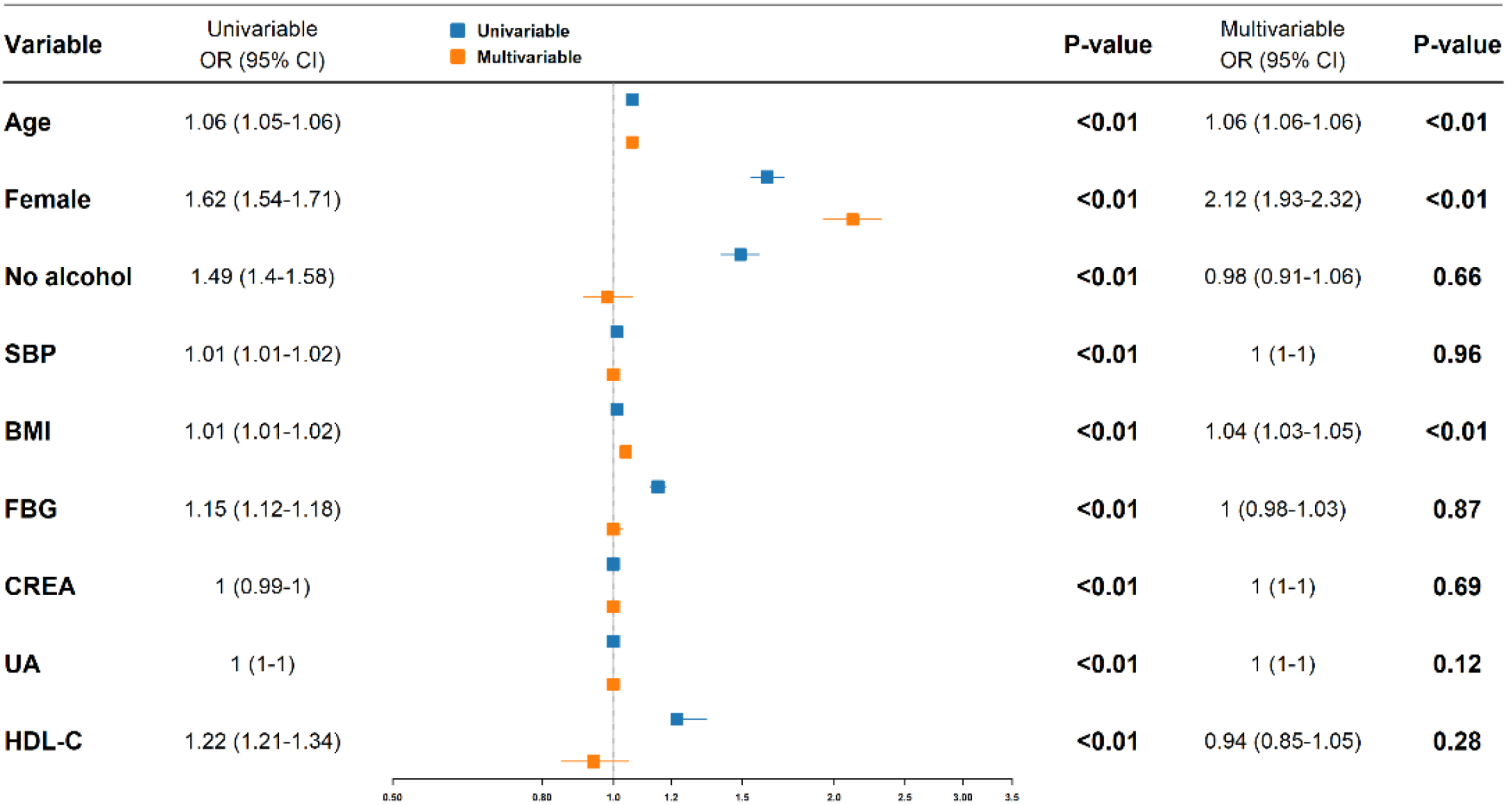
Logistic regression–derived risk factors for thyroid nodules (Forest Plot) OR, odds ratio; CI, confidence interval; Univariable results are shown in blue, and multivariable results in orange. Variables with P < 0.05 in the multivariable model were considered independent risk factors.

In univariate models, age, female sex, no alcohol history, SBP, BMI, FBG, SCR, UA, and HDL-C were all significantly associated with TNs (P < 0.05). Multivariable logistic regression identified three independent risk factors: older age (OR = 1.06 per year; 95% CI, 1.06–1.06; P < 0.01), female sex (OR = 2.12; 95% CI, 1.93–2.32; P < 0.01), and higher BMI (OR = 1.04 per kg/m²; 95% CI, 1.03–1.05; P < 0.01). The findings are illustrated in a forest plot (**Figure 1**).

### 3.5 Simplified three-variable prediction model: development and performance

**Figure 2:**
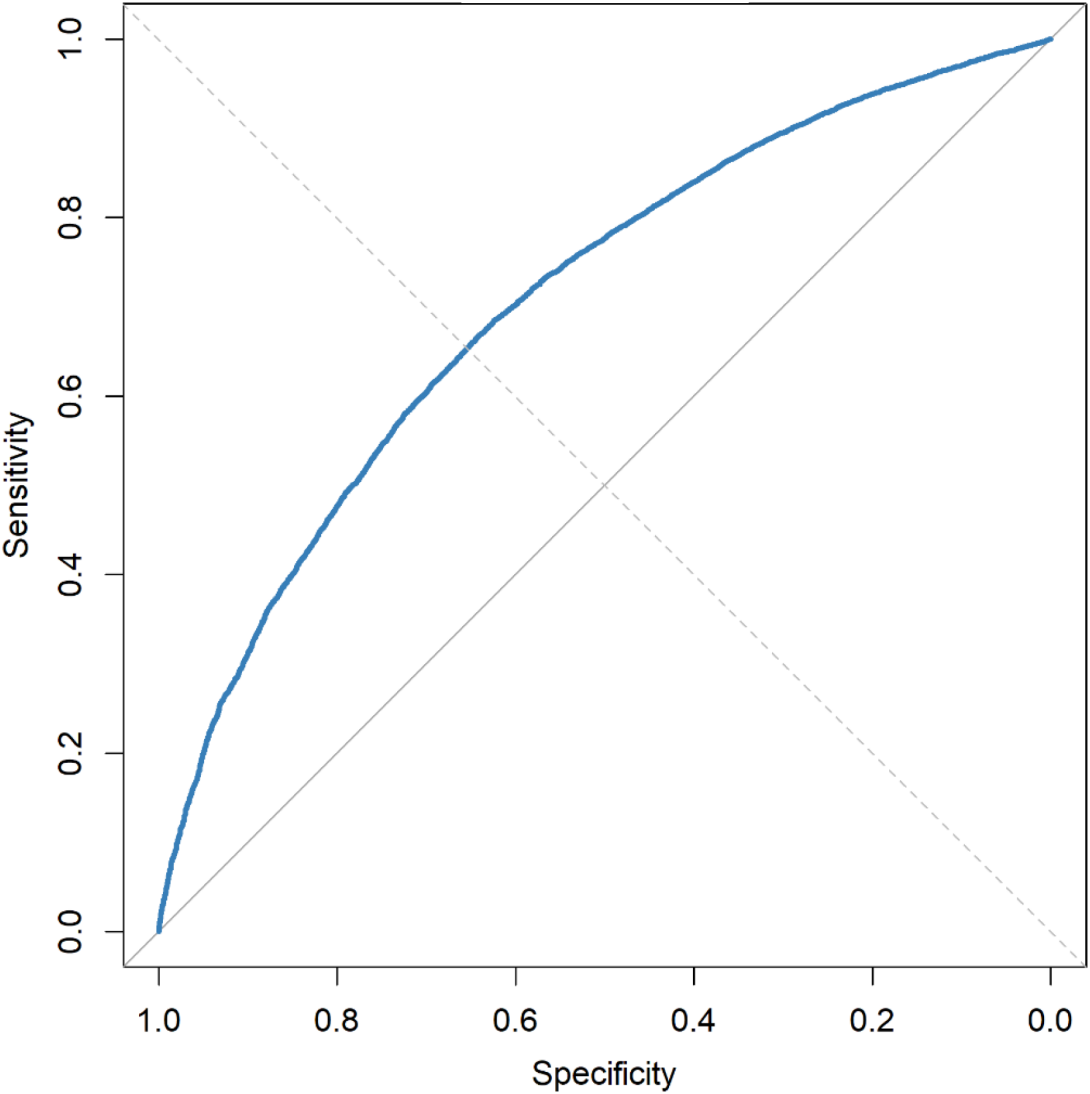
Receiver operating characteristic (ROC) curve of the three-variable prediction model for thyroid nodules. ROC curve of the model including age, sex, and BMI. AUC = 0.706, showing moderate discrimination.

**Figure 3:**
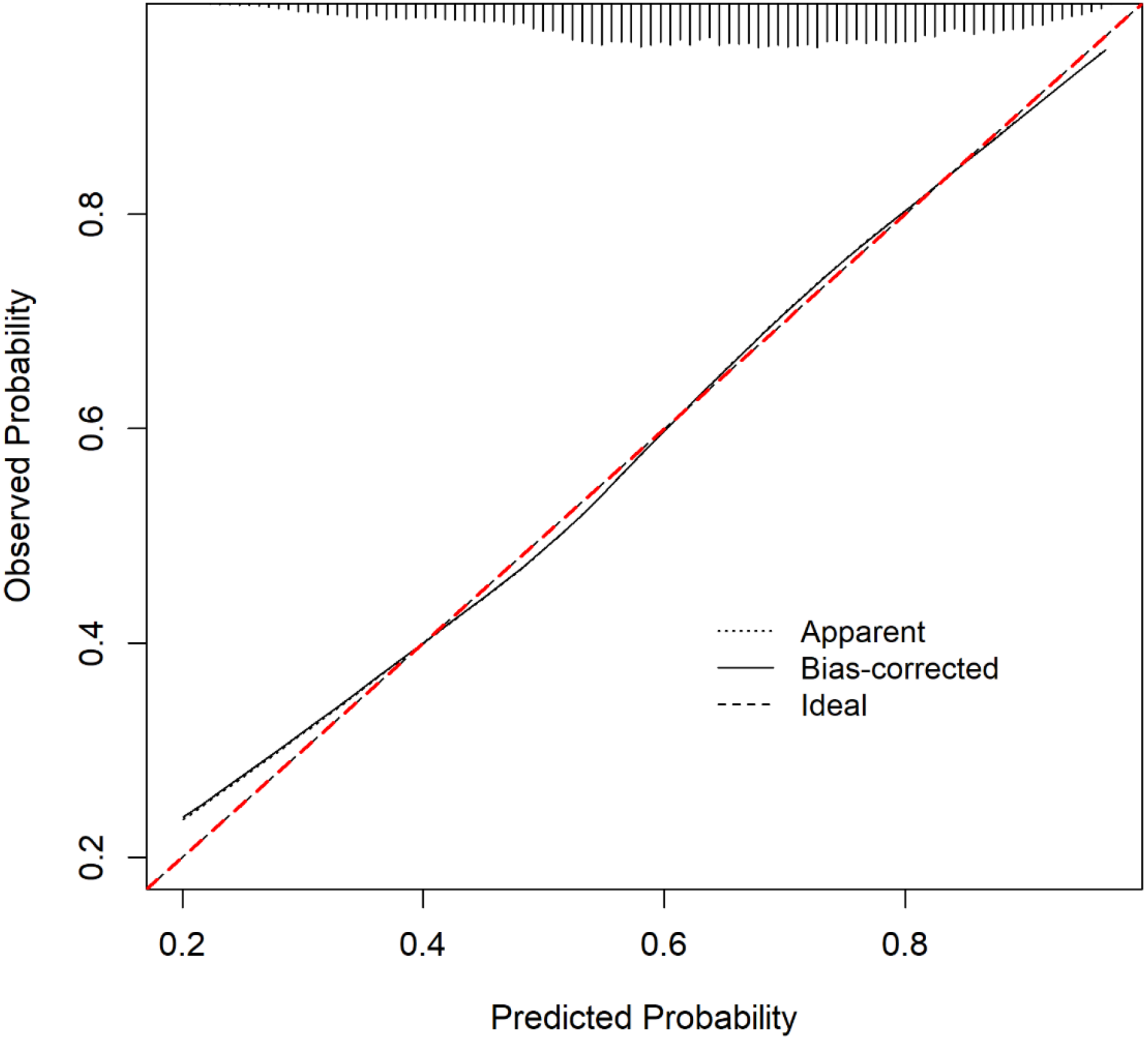
Calibration curve assessing agreement between predicted and observed thyroid nodule risk. Calibration of predicted vs. observed probabilities after bootstrap correction (B = 1000). Bias-corrected line closely follows the ideal line. MAE = 0.007.

A simplified clinical prediction model incorporating age, sex, and BMI was constructed based on the independent risk factors identified in the multivariable logistic regression. When applied to the validation dataset, the model demonstrated moderate discriminatory power with an area under the receiver operating characteristic curve (AUC) of 0.706 (refer **Figure 2**) (24). Calibration performance was evaluated using bootstrap internal validation (B = 1000), and the calibration curve showed good agreement between the predicted and observed probabilities (refer **Figure 3**) (25). The bias-corrected line is closely aligned with the ideal diagonal line, especially within the clinically relevant probability range of 0.4–0.6, indicating excellent calibration. The mean absolute error (MAE) was 0.007, suggesting minimal overall prediction error and high model reliability (24).

## 4 Discussion

In this large cross-sectional analysis of 23,305 adults undergoing routine health examinations, we observed a high prevalence of thyroid nodules (64.7%), which is higher than that reported in many earlier population-based studies but comparable to rates reported in recent ultrasound-based surveys and meta-analyses (6). Several factors may explain the relatively high prevalence observed in this study. First, the study population was derived from individuals undergoing routine health examinations rather than a randomly selected community-based sample. Health examination populations tend to be older and more health-conscious, which has been associated with higher detection rates of thyroid nodules. Second, high-resolution thyroid ultrasonography was routinely used and performed by experienced sonographers, enabling the detection of small or subclinical nodules that might be missed by palpation-based screening or lower-resolution imaging. According to the 2015 American Thyroid Association Management Guidelines, high-resolution ultrasound can detect thyroid nodules in 19%–68% of randomly selected individuals, with higher detection rates observed in women and older adults (26). Third, regional characteristics, including demographic structure and iodine nutrition patterns in Northern China, may also contribute to variability in thyroid nodule prevalence (27).

TN prevalence demonstrated a clear age-related trend, increasing from 38.8% in participants aged ≤30 years to 87.8% in those aged >70 years. Women consistently exhibited a higher prevalence than men across all age groups, with the sex difference most pronounced in the 40–70 age range. These findings align with prior reports and reflect the well-established role of aging and sex hormones in thyroid physiology (27).

Our comparative analysis revealed that individuals with TNs displayed a distinct metabolic profile characterized by higher proportions of overweight/obesity, hypertension, abnormal fasting glucose, hyperuricemia, and elevated creatinine. These patterns are in line with accumulating evidence linking metabolic syndrome components to thyroid nodular disease (28, 29). In particular, obesity has been associated with increased thyroid volume and nodule formation, possibly through insulin resistance and chronic low-grade inflammation (7, 30). Insulin and insulin-like growth factors may exert mitogenic effects on thyrocytes, stimulating proliferation and angiogenesis (31). Hypertension and dysglycemia may also contribute via endothelial dysfunction and oxidative stress, promoting thyroid tissue remodeling (32).

Sex-stratified analyses showed that elevated triglycerides were more prevalent among TN-positive women, whereas smoking and alcohol consumption were significantly associated with TNs in men. Due to the extremely low prevalence of smoking and alcohol use among females in this cohort, reliable assessment of these behavioral factors in women was not feasible, and therefore, no conclusions regarding their association should be drawn. These results suggest potential sex-specific metabolic and behavioral influences on TNs development, likely reflecting differences in hormonal milieu, fat distribution, and lifestyle exposures (33).

Despite the observed univariate associations between TNs and various metabolic indicators, only three factors—age, female sex, and higher BMI—remained independent predictors in the multivariable logistic regression model. This implies that the contributions of other metabolic parameters may be mediated through these primary factors or confounded by them. The biological plausibility of these associations is supported by prior research: aging leads to cumulative oxidative and hormonal changes that affect thyroid morphology, estrogen may stimulate thyroid epithelial cell growth, and obesity-related hyperinsulinemia can enhance thyrocyte proliferation via insulin/insulin-like growth factor 1 signaling pathways (34–36). The non-independence of other metabolic indicators in multivariable analysis likely reflects their secondary or downstream nature relative to these core determinants.

We developed a simplified prediction model for TNs risk based on positive findings from multivariable regression analysis, incorporating age, sex, and BMI. The model demonstrated moderate discriminative ability (AUC = 0.706) and good calibration (MAE = 0.007), indicating solid internal validity. While inclusion of additional variables such as thyroid function tests, iodine status, or inflammatory markers could potentially improve predictive accuracy, the simplicity and accessibility of these three variables make the model particularly suitable for large-scale preliminary risk stratification. Nevertheless, it cannot replace comprehensive, thyroid-specific evaluations, including ultrasonography or detailed biochemical assessments.

Although most thyroid nodules are benign, their extremely high prevalence in health examination populations poses challenges related to over-screening, unnecessary follow-up, and inefficient use of ultrasound resources. In this context, the simplified prediction model proposed in this study is not intended for diagnostic purposes, nor to replace thyroid ultrasonography. Instead, the model is designed as a preliminary risk stratification tool to support large-scale health check-up programs. By relying solely on readily available and cost-free variables—age, sex, and body mass index—the model may help identify individuals with a higher probability of thyroid nodules who could benefit from prioritized or more frequent ultrasonographic evaluation, while potentially reducing unnecessary imaging in low-risk groups. This risk-stratified approach may contribute to more rational allocation of medical resources in population-based health management. Importantly, the simplicity of the model enhances its feasibility and scalability in real-world screening settings, where more complex models may be impractical.

This study makes several important contributions. First, the large and diverse sample, encompassing a wide range of ages, sexes, and metabolic statuses, enables precise prevalence estimates and enhances external validity. Second, beyond well-established factors such as age, sex, and BMI, the study simultaneously evaluated a broad spectrum of metabolic indicators—including hyperuricemia, elevated SCR, and dyslipidemia—allowing for a more comprehensive understanding of their interplay in TNs risk. Third, the construction of a simple three-variable prediction model balances usability with predictive performance, offering a practical tool for large-scale clinical and community screening. Collectively, these findings provide evidence to inform precision screening strategies and early interventions, and lay the groundwork for future longitudinal and multi-center studies aimed at improving TNs prevention and management.

Several limitations of this study should be acknowledged. Most importantly, data on thyroid function parameters, including thyroid-stimulating hormone (TSH), thyroid hormones, thyroid autoantibodies, and urinary iodine concentration, were not available in this health examination database. TSH is a well-established independent predictor of thyroid nodule development and malignancy risk, and iodine status plays a critical role in thyroid morphology and nodular disease. Variations in TSH levels have been reported to be associated with metabolic abnormalities such as obesity, insulin resistance, and dyslipidemia, which are also key components of metabolic syndrome (37). Therefore, the inability to adjust for TSH may have resulted in residual confounding and could have led to partial overestimation or underestimation of the observed associations between metabolic factors and thyroid nodules. Similarly, unmeasured variation in iodine status, which is known to influence thyroid size and nodular formation and may differ across regions and populations, could have modified the relationship between metabolic syndrome and thyroid nodules in this cohort (38). Because this study was based on a retrospective health examination dataset, these variables could not be retrieved.

Additionally, smoking and alcohol history were self-reported, which may have introduced recall or social desirability bias. Finally, our cohort was derived from a single center in northern China, potentially limiting generalizability to other regions or ethnic populations.

## 5 Conclusion

In conclusion, our study demonstrates a high prevalence of thyroid nodules in the general adult population of northern China and identifies age, female sex, and elevated body mass index as the primary independent risk factors. Although other metabolic disturbances—including hypertension, hyperglycemia, hyperuricemia, and lipid or renal abnormalities—were more frequent among individuals with thyroid nodules, their effects appear largely mediated through these core factors.

We developed a practical clinical prediction model based solely on age, sex, and body mass index, which showed moderate discriminatory ability and excellent calibration. This model may facilitate early identification of high-risk individuals and support risk stratification for thyroid nodules in both clinical practice and public health initiatives.

Future studies should aim to validate this model longitudinally across diverse populations and investigate the potential inclusion of thyroid function parameters, iodine status, and inflammatory markers to improve predictive performance and elucidate the mechanistic links between metabolic health and thyroid nodular disease.

## Data Availability

The raw data supporting the conclusions of this article will be made available by the authors, without undue reservation.
